# The Crisis at St. Bartholomew's Hospital

**Published:** 1903-11-14

**Authors:** 


The Hospital.

A Journal of

The Medical Sciences and Hospital Administration.

Vol. XXXV.—No. 894. NOVEMBER 14, 1903.

### The Crisis at St. Bartholomew's Hospital.

The decision of the Governors to adopt the Mansion House plan of reconstruction may prove fatal to the reputation and usefulness of our oldest endowed hospital and medical school. This plan restricts the area of the site to about six acres and perpetuates the old wards which have no cross-ventilation, are imperfectly lighted, and possess few of the requirements of a modern ward. To make it clear what the retention of the patients in the old wards really means, it may be well to briefly state the history of their erection. In 1730 St. Bartholomew's Hospital was rebuilt. The hospital proper consists of four large blocks surrounding a large open square, three of which—the east, west, and south wings—are ward blocks, the fourth contains the Court Room and Administration. The ward blocks contain on each floor two double wards, *i.e.*, back-to-back wards, and the staircase in the centre divides each building into two equal parts. No further details are necessary to demonstrate that old wards of this description which have been built for upwards of 170 years, and have contained patients during the whole, or a greater portion of this time, are altogether out of date, and necessarily unfit for the purposes of a modern hospital with a large medical school. The original plan of the hospital placed the four large blocks surrounding a large open square, and these blocks were so planned as to afford to each a maximum of light and air. The plan as originally conceived was unique, but as the work of the hospital has grown, buildings have been erected in juxtaposition to the wings occupied by patients, whereby the hygienic efficiency of these buildings and also of the site itself has been lowered. At the back of the west wing, for instance, the medical school and other buildings have been placed, with the result that the wards on the lower floors have suffered materially in light and air. These same wards have the further disadvantage that the bodies required for the dissecting room are kept in the basement of the medical school buildings opposite the ward windows, a fact which cannot improve the hygienic condition of these lower floor wards.

It is a grave responsibility for any body of Governors to take upon themselves in these days, to continue to place patients in old wards of this description which are less hygienically sound than they were originally, and further the Mansion House Committee's plan will leave St. Bartholomew's Hospital with a site of about six acres, the area of which is not sufficient for a hospital of 700 beds. The minimum allowance of superficial area for a town hospital is admitted to be one acre per 100 beds, which is little enough in these days when fresh air and abundance of light have been shown to be such all-important factors in the treatment of disease. When, however, it is remembered that it is proposed in addition to place on this site of about six acres a new casualty and out-patient department, a new nursing home, new quarters for the resident medical and surgical staff, an isolation block, and a new mortuary, post-mortem rooms, and pathological department, the decision of the Governors will be generally admitted to be in defiance of all modern, scientific, and hygienic requirements. The result of such a policy as this can only end in one way. When the £350,000 has been expended upon the Mansion House Committee's plan, St. Bartholomew's Hospital within a few years must fall into disrepute and unpopularity as a great public hospital, and the effects upon the prosperity of the medical school cannot fail to be grave and serious.

We publish in another column an important letter which ably states the case against the Mansion House plan from the point of view of those Governors who have apprehended the dangers which at present threaten St. Bartholomew's Hospital. That letter intimates that on the eve of the meeting of the Governors at which the plan was adopted (a report of which appears in another column), the Medical Council of St. Bartholomew's Hospital represented to the Treasurer that the present six-acre site must be increased, and that it was essential that any plan worthy of the hospital and its great traditions must provide for the erection of new wards to accommodate 700 patients. It is stated that these important representations of the Medical Council were kept back from the Governors who voted in ignorance of their existence. If this be so it places a grave responsibility upon the whole of the medical staff of St. Bartholomew's Hospital, who cannot and ought not to remain silent any longer. To longer remain silent must make them responsible for the destruction of this great endowed institution which has so splendid a history, and a record of magnificent work accomplished during the last 700 years. To-day we shall content ourselves with this plain statement of facts because we have confidence in the medical staff. We believe that they will come forward and join hands with Mr. Andrew Motion and other governors and friends of the hospital, who have united on public grounds to do their utmost to save St. Bartholomew's Hospital from the practical ruin with which it is threatened at the moment.

